# The Representation of Agents in Auditory Verbal Hallucinations

**DOI:** 10.1111/mila.12096

**Published:** 2016-02-02

**Authors:** Sam Wilkinson, Vaughan Bell

**Affiliations:** ^1^ Sam Wilkinson Department of PhilosophyDurham University; ^2^ Institute of Psychiatry, Psychology and NeuroscienceKing's College London; ^3^ Division of Psychiatry, University College London

## Abstract

Current models of auditory verbal hallucinations (AVHs) tend to focus on the mechanisms underlying their occurrence, but often fail to address the content of the auditory experience. In other words, they tend to ask why there are AVHs at all, instead of asking why, given that there are AVHs, they have the properties that they have. One such property, which has been largely overlooked and which we will focus on here, is why the voices are often experienced as coming from (or being the voices of) agents, and often specific, individualised agents. In this article, we argue not only that the representation of agents is important in accurately describing many cases of AVH, but also that deeper reflection on what is involved in the representation of agents has potentially vital consequences for our aetiological understanding of AVH, namely, for understanding how and why AVHs come about.

## Introduction

Current aetiological models of auditory verbal hallucinations (AVHs) tend to focus on the mechanisms underlying their occurrence, but often fail to address more fine‐grained aspects of the content of the auditory experience. In other words, they tend to ask why there are AVHs at all, instead of asking, given that there are AVHs, why they have the properties that they have. One such property, which has been largely overlooked and which we will focus on here, is why the voices are often experienced as coming from (or being the voices of) agents, and often specific, individualised agents.

In this article, we argue not only that the representation^1^ of agents is important in accurately describing many cases of AVH, but also that deeper reflection on what is involved in the representation of agents has potentially vital consequences for our aetiological understanding of AVH, namely, for understanding how and why AVHs come about.

We proceed as follows. We begin by outlining the importance that the representation of agents has in AVHs. We explain that there are degrees of agent representation, and distinguish the representation of agency from the representation of agent identity (namely representing a specific, numerically unique agent). We then address, in turn, the representation of agency (Section 2) and the representation of specific agents (Section 3), asking three questions for each: What is it to represent agents and specific agents? Why (in the teleological sense, viz. to what end) do we represent agents and specific agents? When do we represent agents and specific agents in perception? We end by reflecting on the consequences that answers to these questions have for our understanding of AVH.

## 1 The Centrality of Agent Representation in AVH

In this section, we examine the importance of agent representation in AVH. We show how current aetiologies haven't really taken it into account, either as an *explanans* or as an *explanandum*. We then give two reasons for not placing, as many theorists do, the explanatory emphasis on the sensory aspects of AVH.

### 1.1 Current Aetiological Models of AVH

Current aetiological models of AVH tend to address the question of how it is that AVH can occur at all, rather than addressing issues about what is experienced. Such models include ones that take the experience to arise as a result of misattributed inner speech (e.g. Frith, 1992; Fernyhough 2004), intrusions from memory (e.g. Badcock *et al*., 2005), or even just spontaneous activation in auditory cortex (Cho and Wu, 2013). Here we do not want to support or criticise any of these models but focus on an aspect of AVHs that they tend to overlook, namely, the representation of agents.

We want to suggest that the representation of agents needs to be taken into account, not only for accurately *describing* many cases of AVH, but also for helping us understand why they come about. Now, proponents of these models might claim that agents are represented in AVHs because AVHs ‘sound’ like they come from agents or specific agents. Therefore, accounting for the presence of AVHs, qua hallucinatory sensory experiences, has explanatory primacy over the representation of agency in AVH. We want to suggest that an alternative explanatory emphasis is potentially illuminating.

### 1.2 Taking the ‘Voice’ Out of ‘Voice Hearing’

The representation of agents is not only central to describing many cases of AVHs, it is also bound up in how both patients and clinicians talk about AVH.

Just to take a revealing illustration of this; voice‐hearers are often asked (whether in person or in questionnaires), ‘How many voices do you hear?’ A moment's reflection on the meaning of ‘voices’ in this question tells us that this is not asking: ‘How many times do you have the auditory experience of a (sound that is like a) human voice talking to you?’ It is rather asking a question about how many potentially distinguishable individuals the subject hears talking to her. A ‘voice’ here does not mean what it does when you might say, ‘That synthesiser has a setting that produces a voice’ or even ‘James has a beautiful voice’. ‘Voice’, in this context, is bound to a specific agent that can have major significance to the voice‐hearing subject.^2^

These agents can be represented with a differing degree of depth (i.e. the subject's conception of them can be very rich or very sparse), however, many of them persist over time, and sometimes over the course of years or even decades (Hartigan *et al*., 2013). Furthermore, these agents are most often perceived as making coherent communicative speech acts (McCarthy‐Jones *et al*., 2014) and are experienced as interacting with the voice‐hearer. Indeed, over 80% of voice‐hearers report being able to engage in interactive conversations with their voices (Leudar *et al*., 1997; Garret and Silva, 2003). As Hayward *et al*. (2011) note, voice‐hearers often interact with their voices in ways that ‘share many properties with interpersonal relationships within the social world’ (p. 1320). With all of this in mind, it seems clear that the agents represented in experiences of AVH are often central to the phenomena. Indeed we might say that overlooking the role of agency in auditory hallucinations is, in some cases, tantamount to taking the ‘voice’ out of ‘voice hearing’.

### 1.3 Degrees of Agent Representation in AVH

Experience of AVHs can be varied and although the subjective experience of agentive voices occurs in the majority of voice‐hearers (McCarthy‐Jones *et al*., 2014) not every case represents agents in the way that interests us here. There are degrees of agent‐representation in AVH, and it is important to clarify that some auditory hallucinatory experiences are not the subject of this article. We start with the lowest level of agent representation, namely, an *absence* of it, and work our way up to the richest agent representations in AVHs.

#### 1.3.1 Absent Agency

First of all, there are cases of auditory hallucinations that aren't vocal in anyway. Tinnitus is perhaps the most common example (typically consisting of whistling or white noise‐like sounds), but non‐vocal auditory hallucinations can also include the experience of clicks, bangs, horns and, at the more complex end, music (Nayani and David, 1996). However, it's important to note that just because a hallucination isn't voice‐like, it doesn't mean that agency is automatically not represented. Van Putten and Emory's (1973) case report of a military veteran who experienced hallucinations of enemy footsteps would be an example of one such non‐vocal hallucination represented as agentive. Conversely, it is not inconceivable that something could sound qualitatively voice‐like, but with no agency attached to it.

It is not clear to what extent there can be *totally* agentless voices, since, to the extent that something is speech, a communicative intention of some sort is arguably required to disambiguate it. The closest thing we might get are cases where an auditory experience that resembles a human voice is heard, but there is no perceived communicative intention on the part of the hearer. For example, Yoshizumi *et al*. (2004) report experiences of hallucinated screams and groans; in cases of palinacousis, patients can hear the last word or section of a phrase ‘echoed’ (Jacobs *et al*., 1973) and in thought echo, patients hear their thoughts ‘spoken out loud’ a fraction of a second after they have thought them (David, 1994). Nayani and David (1996) report a patient who had the auditory hallucination of ‘the murmur of a crowd’ but with only group agency being represented and seemingly no experience of individual agency.

#### 1.3.2 Agency Without Individuation

Other cases where agency, but not any distinguishable agent, is represented, are in what Dodgson and Gordon (2009) call ‘hypervigilance’ hallucinations. In one case study that they use to illustrate these, a patient who is worried that others think that he is a paedophile hears ‘nonce!’ shouted at him throughout the day. There is no indication that he represents this as coming from a specific person, or that, on two separate instances, he can even ask himself whether it comes from the same or a different person. However, it is clear that there is a communicative intention and that the subject takes the experienced utterance to be directed at him.

#### 1.3.3 Internally Individualised Agency

One step up from this is when the agency that is represented in a given case, is bound to a specific agent in the mind of the voice‐hearer over time. Voices may be associated with agents identifiable by individual characteristics but without any nominative reference—such as those experienced by patients who referred to their seemingly anonymous voices as ‘unknown old woman’ or ‘a man with a deep voice’ in the study of Leudar *et al* (1997) who labels them ‘incognito voices’. Voices may also be given an idiosyncratic or purely internally referenced name. For example, the ‘demonic’ ‘Simon’ and the ‘little boy’ ‘Jeremy’ described by a patient from Knudson and Coyle (2002).

#### 1.3.4 Externally Individualised Agency

Finally, voice hearers sometimes associate their voice experiences with specific identities from the ‘outside world’. These ‘pre‐existing’ identities can include past or current family members, acquaintances or celebrities—or indeed fictional or non‐corporeal individuals from popular culture or religion (Beavan, 2011).

#### 1.3.5 Recap

To sum up, then, we can isolate at least four levels of agent‐representation in AVHs.

1. Absent or functionally absent agency
2. Agency without individualisation
3. Internally individualised identity (anonymous ‘incognito voices’ or those given purely internally generated names)
4. Externally individualised identity (whether fictional or real)

It is important to note that types 3 and 4 make up the vast majority of reported voice hearing experiences (Bell, 2013). Indeed, Corstens and Longden (2013) recently reported that representation of an individualised agent could be formulated in 80% of clinical voice hearers.^3^

### 1.4 Agency and Sensory Quality

Many theorists put minimal emphasis on agent representation in AVHs, perhaps because they assume that the agency and individual agents represented are built out of the auditory qualities, and so the latter is what really needs explaining. For example, Cho and Wu (2013, p. 2) claim that ‘it is simple to explain why the patient misattributes the event to another person: that is what it sounds like’. According to such a view, I hear the voice of, e.g., my stepfather because my auditory experience has the properties that resemble those of my stepfather's voice. Of course, one problem with this is that, even if this is an accurate account, we need to explain why the experience has those properties and not others. Furthermore, in spite of this explanatory challenge, there are some voice‐hallucination experiences for which this cannot be the correct account.

#### 1.4.1 Soundless Voices

Many theorists highlight the fact that AVHs have sensory qualities that are similar to hearing.^4^ Garrett and Silva (2003, p. 445), state that ‘the subjective quality of sensation is a near‐universal feature of auditory hallucinations’ and Cho and Wu (2013, p. 2) note that ‘patients reflecting on the phenomenology of AVH typically report strong acoustical properties that are typically characteristic of hearing another person's voice’. Nevertheless, this experience is by no means universal. For example, Frith (1992, p. 73) maintains that AVHs can involve something more abstract than hearing a voice, ‘an experience of receiving a communication without any sensory component’. Moritz and Larøi (2008, p. 104) suggest that the term ‘voice‐hearing’ may well be a ‘misnomer’, an ‘inaccurate term to express that their cognitions are *not their own*’. The phenomenon of ‘ “soundless voices” have’ been reported as far back as Janet (1889/1973) and Bleuler (1911/1950) and has been widely discussed since (Jones, 2010; Larøi *et al*., 2012). Janet's early account is illustrative:

It speaks to me all the time, said one of them, it told me that it is necessary to go and ask the Pope for a pardon.—Do you know who speaks to you?—No, I don't recognise it, it is not the voice of anybody.—The voice is it far or near?—It is neither far nor near, one would say that it is in my chest.—Is it like a voice?—Not really, it is not a voice, I do not hear anything, I sense that I am spoken to.

In terms of systematic studies, a recent questionnaire study conducted by one our teams, filled in by voice‐hearers from clinical and non‐clinical populations, found a large number of cases where voices where sometimes or always reported as non‐auditory. Here is one example:

It's hard to describe how I could ‘hear’ a voice that wasn't auditory; but the words used and the emotions they contained (hatred and disgust) were completely clear, distinct and unmistakeable, maybe even more so than if I had heard them aurally.^5^

It seems clear that subjects can undergo profound experiences of being spoken to, or communicated with, without the presence of any auditory phenomenology.

#### 1.4.2 ‘Voice’ Hallucinations in Deaf Subjects

Another very powerful reason why it is important not to view AVHs as exhausted by the qualitative features of the auditory experience, and by that same token, to attach greater importance to the representation of agency, concerns data from ‘voice‐hallucinations’ in congenitally deaf patients with psychosis. According to Atkinson (2006, pp. 702–3): ‘Around half of all deaf people diagnosed with schizophrenia report experiencing “voices,” during which they sense someone communicating with them in the absence of any external stimulus’. A qualitative interview by du Feu and McKenna (1999) suggested that the voice hallucinations, even of those born profoundly deaf, had an auditory quality.

However, Atkinson convincingly argues against this, suggesting that this is merely the result of the ‘audiocentric way in which interrogative questions are often framed during research interviews’ (p. 702). In fact, Thacker (1994), approvingly cited in Atkinson (2006), reports that questions about more specific acoustical properties of hallucinated voices are met with responses such as ‘How do I know? I'm deaf!’ Thacker instead reports cases where individuals ‘claimed they were lip‐reading a vague visual percept, but could not clearly see a face, or who felt that they were being finger‐spelled to by a persecutor but were unable to see the hands distinctly’ (Atkinson, 2006, p. 703). She also suggests that, in some cases, the hallucination is best described as a ‘message’ or ‘communication’ hallucination, where the subject can tell what is said, but without there being any sensory component at all (compare with Frith, 1992, p. 73, quoted above: ‘an experience of receiving a communication without any sensory component’).

If it is impossible for congenitally deaf subjects to have ‘auditory’ voice hallucinations, this impossibility could be due to different things (which would correspond to a weaker and a stronger sense of ‘impossible’). In the strongest sense, this impossibility would be due to the inability (perhaps due to recruitment of auditory brain regions for other roles) of congenitally deaf subjects to have *any* auditory experiences *at all*. In the weaker case, this could be due to the mechanisms of AVHs needing to recruit modality‐specific imagery, (or that the AVH is associated with such imagery), where auditory imagery in particular is unavailable to the congenitally profoundly deaf (and so visual and motoric imagery need to be recruited instead). Either way, a purely auditory view of voice hallucinations is deeply damaged by this data from congenitally deaf subjects. The experience of being communicated with by an agent, who is represented at least to some degree, seems central to these cases.

#### 1.4.3 Voices Without Recognisable Sensory Qualities

One further difficulty with the view that voices are identified as agents because they have the sensory properties that resemble those agents, is that many people experience voices for which no clear sensory properties can be assumed. Voices might commonly be experienced as non‐human agents (the voice of spirits, angels, devils and so on) where agent representations are unlikely to be built out of relevant auditory qualities since none exist.^6^ Although one could make a weak claim that some sensory properties are assumed in popular culture (e.g. the ‘booming’ voice of God) this is unlikely to be sufficient to account for the process of inferring such a specific agent and neither does it conform to what we know about these experiences which tend to be idiosyncratic in their sensory qualities (e.g. Dein and Littlewood, 2007).

### 1.5 Our Claim

What we want to claim is the following: We have a natural propensity to represent agents generally, and, in certain circumstances, we have a propensity to represent specific, re‐identifiable agents. Understanding this helps us to explain certain important features of AVH. In Section 2 we examine, first, the representation of agents *generally*. Then, in Section 3, we examine the representation of *specific* individualised agents.

## 2 Representing Agents

In examining the representation of agents, we are going to address three questions:

- What is it to represent agents?
- Why do we represent agents? (What's the use of representing agents?)
- When do we represent agents in perception?^7^

### 2.1 What Is It to Represent Agents?

We might want to rephrase the question as: What is it to represent agents rather than inanimate objects? We represent something as an agent when we represent it as having an informational and a motivational profile (or perspective). Thus, we have a grasp of the fact that it takes the world to be a certain way, and it can want the world to be a certain way. Furthermore, and there is a question about whether this is an additional step, we can be aware that this informational and/or motivational profile can be different from our own. In other words, representing something as an agent is to ascribe it ‘beliefs’ and ‘desires’, where these words are used in the broadest possible sense, where what an agent ‘believes’ is simply how it *takes* things to stand in the world, and what an agent ‘desires’ is simply how that agent wants things to be.^8^ This is often called ‘mindreading’. We will see that there is a narrower sense of ‘belief’ and ‘desire’ where these are taken to be more stable ‘mental states’.

### 2.2 Why Do We Represent Agents?

What is the *use* of representing agents? There are two different, but related uses. One is to predict behaviour. The other is to understand others, and in particular to evaluate them, and hold them accountable. The former is adaptive in any multi‐agent context where it is important for an agent to be able to predict how other agents will behave. Other agents could represent direct threats, insofar as they might attack you, or they could be threats in a less direct sense, insofar as they compete for resources (e.g. mates and food). Thus there might be some levels of deception going on, and here emerges the demand for really quite sophisticated forms of higher‐order mindreading (‘He thinks that I think that he thinks…’). The latter, more normative dimension of mindreading, is clearly adaptive in a society where social norms (positive and negative reactions to acceptable and unacceptable behaviours) are in place as a way of enforcing pro‐social behaviour. For simplicity, we focus on the predictive dimension of agent representation, although the normative elements may well be relevant to interesting features of voice‐hearing (e.g. the sense of guilt or of being judged).

Representing the informational and motivational profiles of others enables us to predict their behaviour through a principle of rationality: we assume that agents (crucially, not necessarily physical ones) will impact upon the world in the service of their goals and take their information about the world into account as a way of doing so. This prediction is achieved (at least in humans) at very different timescales.

Roughly speaking, it is useful to think of three different timescales at which we predict the behaviour of other agents. From the smallest to the largest, these are: (i) goal‐directed bodily actions, (ii) beliefs, desires, intentions (in a narrower sense that will become clearer) and (iii), personalities and preferences. What is interesting is that at all three timescales, there is something like the principle of rational action that guides our predictions, which is, as we mentioned, central to the very concept of an agent (namely, that agents will take into account how they take things to stand in the world and act accordingly in the service of their goals).

At the short timescale of *goal‐directed actions*, the expectation is that action will, first, have a purpose, and, second, be performed as efficiently as possible (Gergely and Csibra, 2003; Jastorff *et al*., 2011). Within the class of short‐timescale goal‐directed actions, it is vital not to overlook communicative actions, such as speech acts. When someone speaks we naturally and intuitively pick up on their communicative intentions, and when we speak we form various assumptions about how our communicative intentions will be picked up on by others.

At the middle‐timescale we get predictions on the basis of beliefs and desires. Here ‘belief’ and ‘desire’ are not meant as the generic term for informational and motivational profiles, but for something less stimulus‐bound than perceptually‐guided goal‐directed action, but still somewhat flexible (more flexible, as we will see, than personality and preference). So, a belief, in this sense, is an informational state that an agent has in various different contexts (contrast with the egocentric informational state that you currently have that concerns your immediate surroundings). This kind of state is supposed to be sensitive to counter‐evidence, but should, for as long as it lasts, guide actions across an agent's life and in various different contexts. Thus, if somebody believes that the Earth is flat, they will be reluctant to sail off into the distance. If somebody believes that certain mushrooms are poisonous, they will refrain from eating them (and prevent the people they care about from eating them too).

At the largest timescale we predict on the basis of personalities and preferences. These are less flexible in the face of contrary evidence, and they enable us to predict by thinking ‘What would John do here?’ There are two very different kinds of representations that drive these predictions. They are: (1) singular representations, and (2) generic representations. The former involves knowing a specific individual well enough to be able to attribute a personality to them. The latter involves having a generic representation of a personality type, or even a social stereotype (‘Loud‐mouthed yuppie’), or even a specific occupation (‘A policeman’), and then recognising that the person here encountered, potentially for the first time, is of that kind. So you might reason: ‘Here is a policeman. How might a policeman behave in these circumstances?’

However, the attribution of a personality to an agent is not sufficient to attribute an identity to specific agents. The latter is what we examine in Section 3.

### 2.3 When Do We Represent Agents in Perception?

There are, roughly speaking, two options here. We might call these options ‘inferential’ vs. ‘intuitive’ views. The inferential view is that we recognise that something looks like an agent and we have learnt how agents behave. In the case of auditory experience, this would involve recognising that something sounds like an agent (usually a human) and therefore must be an agent. The intuitive view is that agent representations are very readily (and, in a sense, non‐inferentially) deployed. In contrast to the inferential view, where this would make no sense, on the intuitive view, it would be possible to experience something as an agent *prior* to being consciously aware of any of its perceivable properties.

Let us make an important side point. You may disagree with our use of ‘inferential’ and ‘intuitive’. In particular, you might think of inferential as something that can be very low‐level. That is a terminological decision. The substantive question is whether the perception, and representation, of agency receives rapid and specialized processing and whether this has consequences for our understanding of AVHs. We think that there are obvious reasons for thinking that it does, and that this makes certain aspects of certain AVHs less mysterious.

#### 2.3.1 Evolutionary Support: Adaptive Hyper‐Active Agent Detection

There is strong experimental and ethnographic evidence to suggest that humans have evolved what Barrett (2004) calls a ‘hyperactive agency detection device’ (HADD). Heider and Simmel (1944) used an animation with moving shapes and found that people are strongly biased towards the attribution of intentional agency when the perceptual data is ambiguous. Guthrie (1980) hypothesised the existence of hyperactive agency detection, on the grounds that it would be highly adaptive; a false positive being generally less costly than a false negative (viz. failing to detect an agent that is there would statistically have graver consequences than mistakenly detecting an agent that is not there). More recently, Atran and Norenzayan (2004) have claimed that ‘natural selection has trip‐wired cognitive schema for agency detection in the face of uncertainty’ (p. 720). Atran (2002) ties this closely to developmental work suggesting an early propensity of humans to attribute agency. We present this sort of data now.

#### 2.3.2 Developmental Support: Early Agency Attributions

Many experiments show that infants from a very young age distinguish humans from non‐humans. From birth, infants demonstrably prefer to look at faces and follow their movement (Morton and Johnson, 1991). Furthermore, they imitate the facial and hand gestures of people (Meltzoff and Moore, 1977). Of course the ability to discriminate humans from non‐humans, firstly, doesn't show that agent representation is going on, and, secondly, doesn't say much about agent‐representation in the absence of recognizably *human* stimuli.

Nevertheless, by the time that infants are 9 months old, they certainly seem to behave in ways that suggest they are attributing agency. They point and request things from others (Bates *et al*. 1975; Butterworth and Grover 1988), and they guide and alter their behaviour towards things based on the behaviours of others towards those things (Meltzoff, 1995).

As for the attribution of agency to things in the absence of specifically human stimuli, Gergely and Csibra (2003) found that 12‐month‐olds ascribed intentions to dots that moved around in a ‘rational manner’. Johnson *et al*. (1998) presented novel objects to 12‐month‐olds, varying the morphology (where the target object either had a face or not) and behaviour (where the behaviour was contingent or non‐contingent on the behaviour of the infant). They found that infants were willing to follow the gaze of something that lacked a face, but which reacted contingently to them. Indeed, there is a lot of evidence to suggest that infants can detect contingency in the first weeks of life (Watson, 1972, 1979; Rovee‐Collier *et al*., 1989).

Of course, early development of agent representation is only *suggestive* of it being ‘intuitive’ (or ‘hardwired’). What further suggests intuitive status is its incorrigibility in the face of counter‐evidence. Consider other intuitive processes, such as those that give rise to visual illusions, such as the Müller‐Lyer Illusion. In the Müller‐Lyer Illusion, two lines that are actually of the same length, look different lengths. What makes this an intuitive process is that no amount of counter‐evidence eliminates the misleading percept. Even if you measure the lengths of the lines, and, on that basis, come to *believe* that they are the same length, they will not cease to be *experienced* as different lengths. The claim that agent representation is intuitive (or hardwired) amounts to claiming that it should be incorrigible in a similar way.

If we take the very same objects used with infants, in Johnson *et al*. 1998, even at their most convincing, they present ample evidence that they are not really agents. As Johnson (2003, p. 553) puts it,

To an adult, it would clearly be an artifact made of synthetic materials with an electromechanical noise generator and mechanically driven movement. If despite this obvious counter‐evidence, it elicited a psychological interpretation in adults, the argument that agent recognition is grounded in a hardwired system would be supported …

The parallels between the adult's attributions and those found previously with infants were striking. Adults used mentalistic language to describe the behaviour in just those conditions that infants followed the object's directional orientation with their gaze. If the object had a face or if it was faceless, but interacted contingently with another agent, adults described it as ‘wanting’ something, ‘looking’ for something, ‘trying’ to do something … If, however, it did not have a face and acted only randomly, adults rarely if ever used mentalistic language to describe its behaviour. This result held regardless of whether the object's behaviour was instantiated auditorily (via contingent or random beeping) or visually (via contingent or random wiggling) (Johnson, 2003, p. 553).

So, the question is whether, in spite of beliefs to the contrary in adults, the ‘illusory experience’ of agency remains. This strongly suggests that it does.

It is interesting that it is contingency of behaviour, rather than specific features of the thing perceived, that plays a more important role in the attribution of agency. Although we are not directly evaluating existing aetiologies, ones that take AVHs to involve self‐produced phenomena whose monitoring has gone awry could easily suggest that the voice's ‘behaviour’ is contingent on the subject's agency, since it is (unbeknownst to the subject) triggered by the subject's agency.

## 3 Representing Specific Agents

Now let's move from agency generally, to the representation of specific agents. Again, we ask the same three questions as before, but this time with regard to the representation of *specific* agents:

- What is it to represent specific agents?
- Why do we represent specific agents? (What's the use of representing agents?)
- When do we represent specific agents in perception?

### 3.1 What Is It to Represent Specific Agents?

What we are talking about here is not just the representation of any agent with current beliefs, desires and intentions, but of a specific, numerically unique individual. Is this merely the representation of the personality? Is this just very fine‐grained mindreading or something different? We think that it is something fundamentally different, but a vital component of healthy social cognition, of successful interaction in a world that is populated by other agents that we encounter and re‐encounter. One way of seeing that this is fundamentally different, is to reflect on the *correctness conditions* of representing another agent in perception. Judging ‘this is *a*’ when encountering someone, is not rendered correct merely by the fact that this person looks just like *a*, or even has a qualitatively identical personality. It is only rendered correct by the fact that the person in question is *a*, namely, the very same individual. Indeed it would be correct even if *a* had radically changed appearance and personality.

What must be added to the capacity to attribute mental states and personalities in order to get from the representation of agents to the representation of *specific* agents? The answer to this, is an ability to re‐identify or (to use a more technical term) ‘track’ individuals. In a paper that puts forward a ‘theory of integrated tracking’ Bullot (2009) presents the example of Mary and her spouse George. Mary successfully tracks George, ‘first as briefly located in her left visual field at t1, then as a voice saying “goodbye” at t2, and eventually as somebody who has arrived back after a day at work and can be perceived in a multimodal experience at t3’ (Bullot, 2009, p. 357). In this case, Mary manages to activate her singular representation for George, the *individual*, only when it is George who is indeed present. And this involves successful re‐identification, successful tracking.^9^

### 3.2 Why Do We Represent Specific Agents?

Why do we keep track of specific individuals? One might think that, like the representation of agents generally, the purpose is to predict their behaviour. However, the answer is a bit more nuanced than this. It is certainly true that successfully re‐identifying someone will enable us to predict their behaviour better. However, that is only because we know their beliefs, desires, personalities and preferences, and, short of any evidence to the contrary, we are bound to assume that these will not have substantially changed. So tracking is very helpful for prediction, but only when coupled with knowledge about the individual, namely, when coupled with the attribution of mental states. Indeed it often tells us when to attribute those properties.

Perhaps one of the key survival functions is simply a matter of security. Associating individuals with risks and being able to infer risk given a particular individual is likely to be a key survival adaptation. One function that might be easy to overlook, and which goes beyond sheer predictability, is the tracking of an individual over time, regardless of how much they may have changed, so that we can reward of punish them for past deeds (‘score‐settling’). Also, it enables us to remember common ground with that individual, what experiences may have been shared, what information would be old news to them and so on. Furthermore, from an evolutionary standpoint, keeping track of individuals would have been extremely important for ‘in‐grouping’ and ‘out‐grouping’, as well of for maintaining a social hierarchy among individuals.

It is also helpful for us to reflect on the sorts of individuals that we bother tracking in this way. Among these are: other human beings that we are likely to re‐encounter (compared to those that we simply brush past on a crowded street), pets, perhaps a very narrow set of inanimate objects that have particular emotional value. In other words, we track things that have some significance to us as individuals, rather than merely as the bearers of certain properties (e.g. where anything else with the relevant property would do just as well—like knives in a drawer). Some of this ‘marking of particularity’ (to use John Locke's phrase) is aided by the very simple linguistic tool of giving it a proper name. Although we haven't the space to go into it here, it might be informative to reflect on the function of giving names to the internally generated identities we mentioned in Section 1.3.3.

### 3.3 When Do We Represent Specific Agents in Perception?

Under what conditions does someone perceive an individual as a previously encountered individual? More relevantly, one can view the same issue in terms of the following question: Under what conditions—if at all—do judgements about identity override conflicting perceptual evidence? Again, as with the representation of agents generally, we have an ‘inferential’ and an ‘intuitive’ option.

On the inferential option we ascribe properties to a currently perceived individual (the way they look, sound, act etc.) and match it to a previously encountered individual. How a judgement about identity could then override conflicting perceptual evidence would have to be through some other non‐perceptual evidence that outweighs it. For example, you might have evidence about an individual's precise location, or you might have been told that they have changed in such and such a way. That way, you could judge that somebody is a certain person without looking (or sounding, or smelling etc.) like that person.

For AVHs, such conflicting information will not be available. Hence (to echo Cho and Wu, quoted above) the subject will represent, for example, her stepfather because it sounds like her stepfather. Of course, then what needs to be explained is how it is that the AVH experience comes to have those auditory qualities. That is precisely the explanatory direction that we are trying to discourage in this article.

On the intuitive option, at least sometimes, a specific agent will be represented without the subject having conscious access, either to any of the properties that have grounded this, or to any other conflicting information (such as the location of the individual). The claim is not that judgements of identity that are evidentially grounded in appearance or personality, or spatiotemporal trajectory, *never* happen, but rather that this is not the only way that they happen. Sometimes, and importantly, these are intuitive. This would mean that sometimes, a judgement about identity will override, or simply bypass, considerations about perceptual information. Why would anyone hold such a view?

#### 3.3.1 An Argument from Delusional Misidentification

One of us has argued (Wilkinson, 2013) that delusional misidentification in the context of traumatic brain injury is not to be understood (as the orthodoxy would have it) as a response to evidence presented to the subject in experience, but rather as a neural disruption to a tracking mechanism (which gives rise to an experience that *already carries* the misidentification). This explains why, for example, the subject with Capgras delusion, can (post hoc confabulated differences in appearance aside) claim (i) that the person currently encountered (who is de facto their father) looks exactly like their father, and (ii) they remember everything about their father. None of the information‐storage is impaired, nor is the information recall. Nor is there anything wrong with the visual perception of faces. However, there seems to be a problem with the sort of linking of information that constitutes successful tracking. The upshot of this is that the subject is presented with someone who they are certain is not their father, even though it looks just like their father (and they admit this). The Fregoli delusion involves the opposite: the claim that a man who looks nothing like the subject's father is the subject's father. Thus, the general lesson is that some tracking (and, ultimately, some identity judgements) are not inferred on the basis of properties.

Could something similar not apply to some AVHs? The representation of the man who abused you as a child is the most active, the one you most fear. Thus, to turn the standard explanatory order on its head: the voice has the properties that it has because it is represented as the voice of a given individual, *rather than the other way around*. For example, it is a deep, gruff voice, because it is represented as my stepfather's voice, rather than the other way around.

#### 3.3.2 Data from Dreams

Another reason to think that identity is something that can be directly experienced, and needn't be inferred from surface properties, comes from dreams. Schwartz and Maquet (2002) compared the content of dream reports with functional imaging during REM sleep (i.e. the sleep stage during which vivid dreams are reported). The central idea is that, in principle, dream features can be mapped onto specific distributions of brain activity. In dreams, it is common for the perceived identity of an individual to come apart radically from the individual's appearance. Consider this dream report: ‘I had a talk with your colleague, but she looked differently, much younger, like someone I went to school with, perhaps a 13‐year‐old girl.’ Or this one: ‘I recognize A's sister […] I am surprised by her beard, she looks much more like a man than a woman, with a big nose’ (both quoted in Schwartz and Maquet, 2002, p. 26 and p. 29). This dissociation between how people look and who they are taken to be, coupled with imaging results, shows, as Schwartz and Maquet neatly put it,

… neuronal processes during sleep can *simultaneously and independently* engage (1) unimodal visual areas underlying the internal generation of a perceptual representation of an individual's face […] and (2) distinct multimodal associative areas in the *temporal lobe* responsible for triggering the retrieval of a familiar individual's identity (2002, p. 26, emphasis added).

To sum up then, it seems plausible that we can, at least sometimes, experience someone as a specific individual in spite of explicit evidence that is either absent or contrary to the fact.

## 4 Additional Explanatory Contributions to an Agent‐based Approach to AVH

Aside from giving a more faithful description of many AVHs, giving the representation of agents due attention has a number of potential explanatory payoffs. We address the payoffs of considering the representation of agency generally, and then the representation of specific agents.

### 4.1 Representation of Agency

If we grant that humans are deeply and innately social beings, and that the representation of agency plays a crucial role in human cognition, then this encourages us to look for explanations at the level of factors that could impact on agent representation.

#### 4.1.1 Integration with Delusions

Given the current scientific theories attempt to explain the symptoms of psychosis, it is not clear *a priori* why both delusions and auditory verbal hallucinations tend to have social content, as opposed to being about any other aspect of the world. For example, in the case of delusions of control, theories have focused on problems with the forward prediction of action as a potential explanation (Frith *et al*., 2000; Frith, 2012) although, in the vast majority of cases, the symptom does not present solely as an experience of actions without intention and without a sense of personal agency but typically involves the belief that they are being controlled by outside agents. Fletcher and Frith (2009) argue for a Bayesian approach to explaining psychotic symptoms within a predictive coding framework that aims to account for delusions and hallucinations within a single model, but it is still unable to explain why prediction errors most typically result in symptoms with social content. As we have noted previously, agent representation seems to be a core human ability that develops from a very early age, and may explain why psychotic experiences are typically social in nature. In Bayesian terms, we have a strong prior probability for interpreting things in terms of agency. Although we have used a Bayesian account of psychotic symptoms as an example here, other accounts—such as Langdon and Coltheart's (1999) deficit account—are equally compatible as ‘second factor’ reasoning. Such biases still need to account for why delusions most commonly have social content.

#### 4.1.2 Social Isolation

Social isolation usually refers to an objective lack of contact with others. Several authors have noted that social isolation may lead to the development of AVH (e.g. Beck and Rector, 2003; van Laarhoven, 1993) with Hoffman (2007) providing the most fully‐developed account. His ‘social deafferentation hypothesis’ proposes that a lack of social stimuli during critical windows in development leads to changes in the brain regions that are associated with social cognition. These changes are analogous to the neural changes observed in individuals who experience ‘phantom limb’ following the loss of a body part. According to Hoffman, these changes can lead to the attribution of social meaning to non‐meaningful events (presumably ambiguous environmental stimuli, such as traffic noise, and internal events, such as inner speech). This erroneous attribution, argues Hoffman, forms the basis of an AVH. Hoffman's hypothesis has much in common with Beck and Rector's (2003) cognitive model of hallucinations. Beck and Rector argue that cognitions are experienced as hallucinations when they pass a perceptual threshold. They identify a number of factors that can make a cognition ‘hot’—such as being emotionally charged—and so exceed the threshold, as well as a number of factors that can reduce the threshold, and so make hallucinations more likely. In line with Hoffman, one of the factors Beck and Rector identify as lowering the perceptual threshold is social isolation.

Putting the emphasis on agent representation, rather than on general capacities of social cognition, we could say that social isolation leads to a bias in the cognitive system toward detecting agents amid non‐agentive environment. On such a view, the perceptual threshold that Beck and Rector (2003) talk about is not solely a lowered threshold of sensory input, but also a lower threshold for the activation of agent representations. This might lead to a heightened sensory *phenomenology*, but this would be consistent with the agent representation being *explanatorily* prior. In other words, you have the experience of someone talking to you because you have represented an agent, rather than because you have a lowered threshold for auditory input.

#### 4.1.3 From Passivity to Alteriority

One aspect of AVHs that many models fail to account for is what might be called ‘alteriority’, namely, the sense that the experience is caused by somebody else. Many theories can account for passivity (the ‘not me’ aspect of the experience) but cannot plausibly account for the step from ‘not me’ to ‘somebody else’, that is, the step from passivity to alteriority. Perhaps these theorists view alteriority as something that is inferred from passivity: The subject reasons, ‘it's not me, therefore it must be somebody else’. However, if we view our basic propensity to represent agents as central to our understanding of some AVHs, then alteriority is built in. Furthermore, this approach has more explanatory potential than the approach where alteriority is inferred automatically from passivity, since it can account for passivity experiences that aren't attributed to other agents, viz. that don't involve alteriority. These phenomena would involve experiences of passivity, but would not trigger (or involve, for whatever reason) our natural propensity to represent agents. The feeling that, for example, an action is not controlled by you will not, in itself, lead you to judge that it is being controlled by somebody else.

### 4.2 Representation of Specific Agents

If we grant that the tracking of specific agents plays a fundamental role in human cognition, then we get a better explanation of why so many auditory verbal hallucinations are experienced as coming from particular agents. More specifically, it might exempt us from having to claim that the agent's identity is inferred from the low‐level properties of the auditory experience. As we've already noted, such a view might claim that, in some cases of AVH, the auditory experience is phenomenologically the way it is because it involves the representation of a particular agent, rather than the other way around, namely the notion that the agent is ‘built up’ from auditory properties. One could further hypothesise that this is more likely to be the case when the represented individual is especially significant to the subject (whether for positive or negative reasons). For example, in cases of bereavement, the subject often hears the voice of the deceased loved one (Keen *et al*., 2013). There are other experiences, too, which are suggestive of a tracking‐first view. For example, there are cases where the presence of the deceased is felt, or footsteps of the deceased are heard (in a way that the subject claims is unmistakably those of the deceased; Carlsson and Nilsson, 2007). With a starkly different valence, voice‐hearers who were victims of childhood abuse can hear the voices of their abusers (McCarthy‐Jones, 2011).

In a related manner, there might be a connection between the agent represented and the distressing nature of the voices. For example, Badcock and Chhabra (2013) show, in a comparison between clinical and non‐clinical voice‐hearers, that the perceived identity of the voice correlates strongly with the distress caused. Furthermore, the fact that a history of childhood sexual and emotional abuse, but not physical abuse, is linked to the likelihood of AVH, may suggest a connection with relationship trauma (Bentall *et al*., 2012).

## 5 Questions and Future Directions

The general shift in focus from the qualities of the experience to the representation of agency also raises some interesting questions, and potential avenues for future research. We examine these now.

### 5.1 Differences in Mindreading or Social Cognition

If the attribution of agency is central to some AVHs, what might this predict about AVHs where the attribution of agency is impaired or different from the so‐called ‘norm’? Are AVHs significantly or illustratively different in their frequency and/or nature in, for example, autistic people or people with specific forms of brain injury? It is worth noting here that we are suggesting that the ability to represent agency is not the same as ‘theory of mind’ .We are arguing that theory of mind (the ability to attribute mental states to others) is a higher‐order function of successful agent representation, as one has to first represent an individual agent to be able to attribute beliefs, desires and intentions to it. However, we would expect that agent representation problems are likely to ‘cascade up’ to theory of mind deficits. Bell (2013) has argued for an agent‐based approach to social cognition in which the representation of internal models of social actors are considered a key component, particularly given the phenomenon of AVHs.

### 5.2 Ego‐Distinctiveness and Agency Models

If agent representation is central to AVHs, then this raises potentially interesting questions regarding ego‐distinctiveness (to what extent the voice is experienced as distinct from the voice‐hearer's own consciousness). On a pre‐theoretical, inferential view, the perceptual or semantic content of the voice might seem ego‐distinct, even radically and distressingly at odds with the intentions of the subject, and *as a result* will be more likely to be attributed to another agent. However, given the centrality of agent representation that we have been gesturing towards, the order of explanation may be more complicated. Ego‐distinctiveness may also reflect the extent to which agency representation processes are affected in the voice‐hearer. Indeed, there are some clues from the clinical literature where need for care varies partly with level of agency representation. For example, voice hearers with a need for clinical care are more likely to attribute their voices to real people rather than non‐corporeal entities (Daalman *et al*., 2011) and they are also more likely to experience their voices as ‘malevolent, omnipotent, intrusive, dominant, and coercive’ (Johns *et al*., 2014).

### 5.3 Agency and the Perceived Reality of Voices

Finally, one might wonder how the representation of agency ties into epistemic aspects, i.e. concerning the belief that the voices are real. Again, this can be separated into the generic representation of agency, and the representation of specific agency. Thus one could explore whether subjects who attribute some (non‐identity‐specific) agency are more likely to view their voices as real. Alternatively one could explore whether subjects who represent specific agent identities (whether created or real) are more likely to view their voices as real. Then, given an established relationship between the perceived reality of the voices and (in negative voices) the extent to which they are experienced as distressing or troubling, this could suggest potential therapeutic avenues.

## 6 Conclusion

We have tried to shine the spotlight on an aspect of voice‐hearing that has either been overlooked entirely, or has not been given its due importance (both descriptive, and explanatory). How different existing aetiologies can accommodate agent representation in a way that lets it play the explanatory role that we have in mind is an open question. However, it seems clear that, at first glance, the three families of model that we mentioned earlier (inner speech‐based, memory‐based and spontaneous activation models) have different prospects for being able to do this. Memory‐based models seem the best equipped to incorporate agent representation in the relevant way, since memories are very often populated by abstract multimodal agent representations. Conversely, spontaneous activation models seem to be less well equipped, at least insofar as they currently appeal to spontaneous activation in primary auditory cortex (Cho and Wu, 2013). How well inner speech models fare in this regard will, in part, be determined by how happy we are to accept that inner speech can involve representations of other agents.^10^

## Footnotes

1 We mean ‘representation’ in the broadest sense, namely, that in virtue of which a mental state is about (or purportedly about) something. Thus, against more stringent uses of ‘representation’, you can have singular representations (ones that are only about one unique thing) as well as general ones (for properties and kinds). In a related fashion, you can also have representations that have no ‘descriptive content’, that are ‘non‐descriptive modes of presentation’ of individuals.

2 Using this sense of ‘voice’, it is not a contradiction to say: ‘The voice [the agent] has changed its voice [the sound]’.

3 Many thanks to an anonymous referee, for drawing our attention to this recent paper.

4 Of course, these theorists might be stipulating that by AVHs, they only mean those experiences that have auditory phenomenology.

5 This questionnaire was conducted online as part of the Wellcome Trust funded project ‘Hearing the Voice’ and ethically approved by the Durham University Philosophy Department Research Committee. Participants were asked to provide free text responses to several questions about voices and voice‐like experiences.

6 We would like to thank an anonymous reviewer for this valuable point.

7 The reason why we are focusing on perception (perceptual experience) rather than thought is, firstly, because we are concerned with AVH, which is a perceptual experience. Secondly, we take this question to divide relevant empirical options in a way that agent representation in thought doesn't.

8 An important question in philosophy concerning whether intentions should be added to beliefs and desires an irreducible mental state, or whether intention is reducible to desire and means‐end belief.

9 The point that representing personality and representing identity (a specific individual) are different can be put in logical terms. Personality is a predicate. It is something that can be said of something (or ‘an individual’ in the bare, logical, sense). Thus two things can have the same personality, and one thing can change its personality. An individual is not a predicate, it is something *to which* you *attribute* predicates. It is, in the formal language of first‐order predicate logic, re‐identification is of the form ‘a = b’ not of the form ‘Fa’. Re‐identifying a specific individual is, therefore, not predicative. It involves drawing a link between two individuals, rather than attributing a property to an individual.

10 McCarthy‐Jones and Fernyhough (2011), in a study examining the varieties of inner speech, suggest that inner speech contains representations of other agents 25.8% of the time.
